# Identification of general characteristics, motivation, and satisfaction of internet-based medical consultation service users in Croatia

**DOI:** 10.3325/cmj.2011.52.557

**Published:** 2011-08

**Authors:** Ivana Klinar, Ana Balažin, Bruno Baršić, Hrvoje Tiljak

**Affiliations:** 1 Pliva Croatia Ltd., Zagreb, Croatia; 2 Health Center Zagreb, Zagreb, Croatia; 3 Dr Fran Mihaljević, Hospital for Infectious Diseases, Zagreb, Croatia; 4 University of Zagreb School of Medicine, Zagreb, Croatia

## Abstract

**Aim:**

To identify users’ reasons to look for physician consultation on the internet instead of visiting a physician and to explore their general characteristics, motivation, and satisfaction with internet medical consultation service ‘Your Questions.’

**Methods:**

Users of a free internet medical consultation service ‘Your Questions’ (*www.plivazdravlje.hr*) were invited to participate in a web-based survey designed to explore their general characteristics (age, sex, etc), reasons for using the service, the nature of their health problem or question, and their satisfaction with the service. Respondents were divided into two groups: users who consulted an internet physician only (Group I) and users who used internet consulting before or after visiting a physician (Group II).

**Results:**

The response rate was 38% (1036/2747), with 79% female respondents. A fifth of the respondents (21%) consulted an internet physician only (Group I). Multivariate analysis revealed that the respondents in Group I were younger (median 24 vs 28 years in Group II), more interested into questions about pregnancy (odds ratio [OR], 1.984; 95% confidence interval [CI], 1.203-3.272), more often embarrassed to talk to a physician in person (OR, 1.828; 95% CI, 1.119-2.989), and more motivated to protect their privacy (OR, 1.727; 95% CI, 1.252-2.380). They also had greater satisfaction with the service (77% vs 60%, *P* < 0.001).

**Conclusion:**

The factors associated with the use of internet-based medical consultation services were younger age, need for privacy protection, avoidance of embarrassment at the physician’s office, and having a question related to pregnancy. This reveals the internet medical consultation service as a useful health promotion supplement that is particularly applicable for the population of young adults.

Through television, magazines, and the internet, patients are today exposed to various medical information resources (1,2). The internet has profoundly changed communication patterns, ever since its introduction in the late 1990s (3). The changes have also been influencing the health care area: a large number of internet users search for answers to numerous health questions through internet portals, which increasingly offer advice on healthy lifestyles and medical issues (4,5).

Another challenge evident in many European countries, as well as in Croatia, is the lack of medical workforce. In Croatia, health care services are covered by compulsory health insurance (6). During 2005 and 2006, there was a need for 171 additional general physicians (7), 58 gynecologist teams, and 85 pediatricians in the primary health care network (8). Under these circumstances, a reliable internet health service could serve as a useful supplement to public health care service (1,2,4,9). It could help unload the primary care system in the area of provision of medical information to the patient.

Internet medical services enable communication with a physician by either a web-based messaging system or e-mail. Reported reasons for sending an enquiry to an internet physician are privacy protection and convenience issues or frustration and disappointment with a previous physician (10). Although numerous specialized services are established to offer a web-based consultation between laymen and medical professionals, only a few services provide general medicine consultations (10,11), addressing various specific medical fields such as cancer, diabetes, depression, phobia, pain, and diet (1,11,12). This is especially the case in the region where Croatian language is used (13,14).

One of these services in Croatia is the internet-based medical consultation service ‘Your Questions,’ on the general medicine portal *www.plivazdravlje.hr*, which has been active for a decade. PLIVA Croatia Ltd, within its public health program, launched this interactive service to provide users with the possibility to better understand specific medical conditions and symptoms, as well as to increase their adherence to specific recommended therapies and interventions by their health care providers.

The portal consists of virtual encyclopedia, actual articles, news, and ‘Your Questions’ service with ten predefined categories: Woman Health, Sexually Transmitted Diseases (STD), Pregnancy, Male Health, Mental Health, Cardiovascular Diseases, Gastroenterology, Neurology, Pediatrics, and Others. The service provides free of charge web-based asynchronous consultation with family physicians and specialists who answer users’ questions regarding health, symptoms or diseases, and give basic information about the treatment options the users have already received.

The interactive internet-based medical consultation service ‘Your Questions’ has been used extensively since its launch, with an average service load of 30 questions a day. This fact intrigued us to find out more about the users and their reasons for using the internet for medical consultations. Specifically, we wanted to explore whether the users visited their physicians or only consulted internet physicians, as well as what the exact factors associated with seeking medical consultation through internet were. We also wanted to explore the general characteristics of the internet medical consultation service users, in order to find out what motivated them to post questions to an internet physician and how satisfied they were with the consultation they received. We also expected that the results of our survey will give us the basis and guidance for service improvements.

## Methods

### Internet-based medical advice

During the entire 2007, we analyzed the number of posted questions in different predefined ‘Your Questions’ categories of the *www.plivazdravlje.hr* medical portal. We also analyzed the frequency of questions answered by general physicians.

### Physicians

In 2007, 9 general physicians and 13 specialists provided medical consultations at *www.plivazdravlje.hr* on a part-time basis. All physicians engaged in the service were practicing physicians providing state of the art medical information, using generic names of the drugs according to the current specific guidelines and treatment algorithms and without recommending any specific drug treatment to avoid any possible conflict of interest. Physicians received financial compensation for their time spent in answering the users’ questions in an amount that represented the fair market value for their services.

General physicians answered the questions either directly to users’ e-mail addresses or by forwarding questions to specialists and then upon receiving specialist’s answers, sending them directly to users’ e-mail addresses. All the answers were signed either by a general physician or a specialist. In that way, the portal protocol simulated the existing health system in Croatia, being based on a network of general physicians who serve as gatekeepers and coordinators for all services provided by specialists in outpatient and/or inpatient clinics. The answers were mostly personalized, in some cases standardized, but the language was always as simple as possible. Each message was without a definitive diagnosis, a diagnostic evaluation, or some specific recommended therapies, and the majority included health and lifestyle promotions as well as advice for further consultation with the treating physician if needed. In the disclaimer, it was pointed out that the consultation and answers were not substitutes for direct medical advice by their physicians, and that there were certain limitations of such communication. The possibility of using e-mail addresses in surveys was mentioned on the portal and was used for the web-based survey in 2007.

### Web-based survey

In July 2007, all users who had used the internet-based medical consultation service ‘Your Questions’ during the first six months of 2007 were invited to take part in a web survey. Each user received an e-mail message containing an invitation letter with a hyperlink to the web-based survey that had been created especially for the purpose of this study. No follow-up reminder emails were sent. The survey was created using the Hypertext Preprocessor script language. All questions were listed on a single web page, and answers could not have been submitted unless all questions were answered. The estimated time needed for answering and submitting the answers was 5-10 minutes. The answer formats included multiple choice questions with one answer or multiple answers, and open-ended questions. The survey consisted of 20 questions related to users’ basic characteristics, reasons for using the service, health related issues that motivated them to use the service, visits to the physician due to the medical issue in question, and satisfaction with the answer they had been given (web-extra material) (web extra material 1). Medical fields were initially analyzed according to the predefined ten categories. After having collected the data, we grouped the answers into three categories based on the obtained frequencies: pregnancy as a separate condition and not considered an illness, sexually transmitted diseases/women’s health, and all other conditions.

To assess the factors associated with internet consultations only, we divided the three categories of users into two groups: Group I – users consulting only an internet physician and Group II – users combining the internet physician consultation with visits to the physician’s office. Answers to open-ended questions are not presented in this article.

### Statistical analysis

The answers on questions are represented as continuous variables (age) and categorical variables. We calculated median and 25th and 75th percentile for continuous variables, and frequency and percentages for categorical variables. Differences in continuous variables between the groups of interest were assessed by the Wilcoxon rank-sum test, and for categorical variables with the Fisher exact test.

Logistic regression analysis was performed to better estimate factors independently associated with the consultation of an internet physician and a visit to a physician. The variables that entered the logistic regression model were selected on the basis of bivariate analysis comparing the two groups. All the variables significant at the threshold of significance set at 0.05 were entered into a multivariate model. The assessment of how the statistical model fitted was done applying the Hosmer-Lemeshow goodness-of-fit test. All *P* values are two-tailed, with significance set at <0.05. For all the analyses, we used SAS 9.2 statistical software (SAS Institute Inc., Cary, NC, USA).

## Results

In 2007, 6402 question were received using the internet-based medical consultation service ‘Your Questions’ on the *PLIVAzdravlje* portal. Most of the questions were in the categories of Women’s Health, 1298 (20%); STD, 821 (13%); and Pregnancy, 816 (13%). In the remaining categories, the percentages of received questions were as follows: Cardiovascular Diseases, 493 (8%); Men's Health, 431 (7%); Gastrointestinal Conditions, 376 (6%); Psychiatry, 380 (6%); Neurology, 374 (6%); Pediatrics, 323 (5%); and Others, 1090 (17%). After the triage by the general physician, only 14% of all questions were forwarded to specialists. Out of 2747 users who used the service in the first six months of 2007, 1036 (38%) responded to the web-based survey. Group I consisted of users consulting an internet physician only and Group II of users who combined the internet physician consulting with a visit to a physician. Most participants (822/1036, 79%) belonged to Group II (32% visiting the physician before consulting on the internet, 30% visiting the physician before and after consulting on the internet, 18% visiting the physician after consulting on the internet), while only 214 (21%) respondents consulted only an internet physician.

Most of the respondents were women (814/1036, 79%) and 52% (541/2036) had college or university degree. Also, most were from Croatia (72%), while the rest were from the neighboring countries, 16% from Bosnia and Herzegovina, 5% from Serbia, and 7% from other countries (Table 1).

**Table 1:** Basic sociodemographic data and comparison between the respondents who consulted internet physician only (Group I, n = 214) and those who sought internet advice in addition to visiting a physician (Group II, n = 822)

	No. (%) of respondents in			*P*
Characteristic	Group I	Group II	overall	
Age (years), median (range)	24.0 (21.0-29.0)	28.0 (24.0-35.0)	27.0 (23.0-33.5)	<0.001*
Sex:				
male	46 (21.5)	176 (21.4)	222 (21.4)	0.979^†^
female	168 (78.5)	646 (78.6)	814 (78.6)	
Country:				
Croatia	165 (77.1)	585 (71.2)	750 (72.4)	0.582^†^
Bosnia and Herzegovina	30 (14.0)	134 (16.3)	164 (15.8)	
Serbia	7 (3.3)	46 (5.6)	53 (5.1)	
Macedonia	4 (1.9)	20 (2.4)	24 (2.3)	
Slovenia	1 (0.5)	3 (0.4)	4 (0.4)	
other	7 (3.3)	34 (4.1)	41 (4.0)	
Education:				
elementary school	5 (2.3)	7 (0.9)	12 (1.2)	0.228^†^
high school	99 (46.3)	384 (46.7)	483 (46.6)	
college degree	42 (19.6)	142 (17.3)	184 (17.8)	
university degree	68 (31.8)	289 (35.2)	357 (34.5)	

*Wilcoxon rank-sum test.

†χ^2^ test.

Respondents in Group I were significantly younger (24.0 years; 25th and 75th percentile 21.0-29.0) than those in Group II (28.0 years; 25th and 75th percentile 24.0-35.0) (*P* < 0.001). Other characteristics, such as sex, education, and country of origin were not significantly different between the two groups (Table 1).

Respondents asked questions mostly for themselves (80%), while 11% of them asked questions on behalf of their children and 10% on behalf of a partner or a spouse. Generally, in both groups, when asked about the main reasons for posting a question, most of them reported the convenience of the continuous information availability on the medical portal: 64% in Group I vs 59% in Group II (*P* = 0.144).

A significant difference between the groups was related to privacy protection and embarrassment. In Group I, there were more respondents who wanted to protect their privacy (56% vs 17%) and have the opportunity to ask a possibly embarrassing question than in Group II (37% vs 6%,; *P* < 0.001). Twenty percent of respondents in Group II posted questions because their treating physician did not have the time to answer their questions in an adequate way (*P* < 0.01) (Table 2).

**Table 2:** Reasons for consulting a physician on the internet-based medical consultation service portal ‘Your Questions.’ Group I (n = 214) – respondents who consulted only internet physician; Group II (n = 822) – respondents who sought internet advice in addition to visiting a physician

	No. (%) of respondents in			
Question	Group I	Group II	overall	P*
On whose behalf the respondents submitted the question:^†^				
themselves	177 (82.7)	650 (79.1)	827 (79.8)	0.173
partner or spouse	28 (13.1)	78 (9.5)	106 (10.2)	0.122
child	21 (9.8)	95 (11.6)	116 (11.2)	0.471
parent or other relative	15 (7.0)	82 (10.0)	97 (9.4)	0.185
someone else	10 (4.7)	31 (3.8)	41 (4.0)	0.547
Reasons for posting the question:^†^				
no time to visit a physician	12 (5.6)	30 (3.6)	42 (4.1)	0.196
protecting privacy	120 (56.1)	305 (37.1)	425 (41.0)	<0.001
continuous availability of information	137 (64.0)	481 (58.5)	618 (59.7)	0.144
do not want to wait at the physician’s office	21 (9.8)	81 (9.9)	102 (9.8)	0.986
embarrassed to ask a physician	36 (16.8)	52 (6.3)	88 (8.5)	<0.001
physician does not have time to answer my questions	26 (12.1)	168 (20.4)	194 (18.7)	0.006

*χ^2^ test.

†Multiple answer question.

In both groups, most common reasons for a consultation were STDs and gynecological concerns, 36% in Group I and 35% in Group II. Questions associated with pregnancy were more common in Group I – 34/214 (16%) vs 68/822 (8%) (*P* < 0.01) (Table 3).

**Table 3:** Characteristics of health problems among respondents who consulted only an internet physician (Group I, n = 214) and who sought internet advice in addition to visiting a physician (Group II, n = 822).

	No. (%) of respondents in			
Question	Group I	Group II	overall	P*
Medical category to which the question pertains				
sexually transmitted disease and gynecological problems	77 (36.0)	290 (35.3)	367 (35.4)	0.002
pregnancy	34 (15.9)	68 (8.3)	102 (9.8)	
other medical categories	103 (48.1)	464 (56.4)	567 (54.7)	
Characteristics of health problem:^†^				
present symptoms of illness	114 (53.3)	320 (38.9)	434 (41.9)	0.002
search for a second opinion	23 (10.7)	275 (33.5)	298 (28.8)	<0.001
wish to get more information	54 (25.2)	312 (38.0)	366 (35.3)	<0.001
more knowledge about particular drug	23 (10.7)	75 (9.1)	98 (9.5)	0.470
more knowledge about particular diagnostic procedure	16 (7.5)	118 (14.4)	134 (12.9)	0.008
more knowledge about treatment possibilities	40 (18.7)	192 (23.4)	232 (22.4)	0.145

*χ^2^ test.

†Multiple answer question.

When asked what motivated them to post a question to an internet physician, 53% respondents in Group I and 39% in Group II (*P* = 0.002) reported that they wanted to find out a possible diagnosis for the present symptoms; 25% in Group I and 38% in Group II (*P* < 0.001) wanted additional medical information about a certain medical condition; and 36% in Group II and 11% in Group I (*P* < 0.001) sought a second opinion. The number of respondents who sought information about the already established medical diagnosis or more knowledge about a particular drug, diagnostic procedure, or treatment possibilities was not significantly different between the groups (Table 3).

The explanatory variables identified by bivariate analysis, comparing two groups of interest that entered the logistic regression model, were important reasons for preferring consultation with an internet physician to visit to a physician, protecting privacy, embarrassment about visiting a physician, age and diagnosis of conditions, pregnancy and other categories vs gynecological problems and STDs. Questions related to pregnancy were more common in participants who consulted only an internet physician, while differences between two groups were less pronounced for STD and gynecological reasons. This is why we chose STD and gynecological reasons as referral group against which other reasons were compared. The model was well fitted. Interactions between the variables and multicollinearity were not assessed. The generalized and adjusted coefficients of determination in the final model showed that the model had very good quality prediction, ie, satisfactory explanatory value (c = 0.692).

The factors associated with consultation with an internet physician exclusively and posting a question to the internet-based medical consultation service were younger age, protecting privacy, and embarrassment about visiting a physician. The increase in age for one year decreased the probability of consulting the internet physician only by 5% (Table 4). The questions related to pregnancy issues were almost two times more frequently associated with consulting only the internet physician than STDs and gynecological problems (Table 4). Overall, 64% of respondents were satisfied with the answers they had received from the internet medical consultation service, with greater satisfaction in Group I – 77% vs 60% (*P* < 0.001) (Table 5).

**Table 4:** Characteristics of respondents who consulted only an internet physician on the internet-based medical consultation service ‘Your Questions*’* in comparison with respondents who sought internet advice in addition to visiting a physician

Effect	Odds ratio (95% Wald confidence limits)
Age of the user (per year)	0.951 (0.930-0.973)
Protecting privacy as a reason for seeking internet advice	1.727 (1.252-2.380)
Embarrassment from visiting a physician as a reason for seeking internet advice	1.828 (1.119-2.989)
Presence of symptoms as a characteristic of health problem for seeking internet advice	1.371 (0.994-1.891)
Medical reasons for seeking internet advice:*	
other categories vs sexually transmitted diseases and gynecological problems	1.049 (0.741-1.485)
pregnancy vs sexually transmitted diseases and gynecological problems	1.984 (1.203-3.272)

*Referent category: sexually transmitted diseases and gynecological problems.

## Discussion

Our study showed that the factors associated with the use of internet-based medical consultation services in Croatia were younger age, need for privacy protection, avoidance of embarrassment at the physician’s office, and having a question related to pregnancy.

Our survey did not cover Croatian citizens in general but a limited sample of internet users who knew about the ‘Your Questions’ service; consequently the results of our study are not representative of the whole population of internet users in Croatia. Our web survey had a response rate of 38%, which corresponded with the response rates of similar surveys (10) although we did not use a follow-up. We consider our results and analysis to be of interest to all involved parties: patients/internet users, health care providers and service providers, as the results identified some users’ reasons and characteristics which led them to post a question to the internet-based medical consultation service.

According to the demographic characteristics, our respondents belong to the group of typical internet health information users, as specified by Delić (13): younger women with at least college education who used the internet to receive quick professional advice from a general physician or specialist. Other studies also found that female patients were more frequent users of medical services and in general showed greater interest in their health and the health of their families (15-18). The most frequent fields of interest to our patients – pregnancy, women health, and STDs – are also in line with the findings that women's health is on the top of the list of medical information searched for on the internet (15). Pregnancy, however, should be considered separately since is not a disease and pregnant women tend to have a lot of questions. Interestingly, we found that some of the users in Group I specified that they wanted a second opinion, which would imply that they had already consulted a physician or another source of information (13).

It is a well-known fact that physicians are only able to devote a certain amount of time to each patient, leading some of them to seek additional information on the internet (10). With an internet consultation service such as ‘Your Questions,’ patients can have access to professional and up-to-date medical information that can complement and enhance prior visits to their physician (10,19). This is especially the case in Croatia, considering the shortage of physicians within the primary health care sphere (6-8,20). For example, in 2004, there were 213 gynecologist teams working on a full time basis, with each of them taking care of 6000 women on average (6). Some of the evident downsides of this are longer waiting times for examinations, work overload for physicians, and not enough time for one-on-one patient consultations. Because of the lack of physicians (6-8,20), in the Netherlands there has emerged a new profession of health educators (21) and in some cases educated nurses may have a greater role in providing materials and guiding people to quality sources of medical information on the internet (22). The option of providing medical advice via the internet can be very helpful in the case of our chronic lack of primary health care physicians such as gynecologists. Croatian health care system should pay special attention to the population seeking this type of advice and provide them with medical information on the internet, where they have enough opportunities to ask questions freely.

Our study revealed that continuous availability of information, protection of privacy, time management, and embarrassment were the reasons for posting questions on the medical portal. Young people were more likely to cite embarrassment and protection of privacy. Such results correspond to the observation that young people tend to avoid visiting a physician whenever they can (18,23). It has also been shown that young people usually obtain information through a search engine (24), but for such search of health-related information one should be educated, trained, and have critical thinking skills (18,25). Forty-two percent of our respondents were motivated to consult an internet physician because they had experienced certain symptoms. This observation may signal that a certain proportion of internet users could underestimate their health problems and/or postpone their visit to physicians when needed. This raises the question of the potentially dangerous effect of introducing internet consultations. If the internet would become a substitute for direct consultation with physicians, it could lead to severe mistakes (9,19).

According to some findings, most patients want to be fully informed about their health issues and be able to take part in the treatment decision-making process (9,10,12). This is especially the case with younger patients and patients with a higher socio-economic status (10,19). In these instances, physicians still remain the primary source of health information (1). Therefore, it has been suggested that physicians should have the role of coaching and guiding patients toward reliable medical resources on the internet (1). Eysenbach suggests that health professionals could also educate and train patients on how to ‘filter’ information (26). On the other hand, health professionals themselves should be trained in this (27). In order to minimize the possible risks of misdiagnosis and a negative impact on the physician-patient relationship, many authors agree that the internet should only be a supplement to a regular health care system (1,2,4). The role of the ‘informed patient’ is still under investigation to elucidate controversies in the patients’ information-seeking behavior on the internet (28).

In our study, respondents who consulted an internet physician only were more satisfied with the answer than those who also visited a treating physician. This can be explained by the advantages of the internet such as not having to commute to reach the medical facility, wait for consultation, see an overloaded and dissatisfied physician, and experience potential embarrassment. This should be considered when developing future health care models. One of such models could include the possibility for patients to consult their physician via e-mail. This already seems to be a plausible proposition since a considerable number of adults search for medical information on the internet – 80%-85% in the USA (24), 60%-80% in Northern Europe, 40% in Eastern Europe, and 20%-30% in Southern Europe (9,29,30).

Strategy for the health care reform should include new ways to introduce medical consultation into the national health care system, which could save physicians’ time. Compensation for e-mail service to physicians should be established (31) and incorporated into the national health insurance policies. Our study revealed that almost 90% of all questions posted by users can be answered by general physicians, which would result in savings of both time and financial resources. The financial aspect is definitely worth of mentioning. It has been shown in Great Britain that self-diagnoses by means of the internet could save 44 million pounds a year. Due to information obtained through the National Health Service Web site, patients decided not to make an appointment at a physician's office (32). We can conclude that savings in the time of a financial crisis are more than welcome.

The model of posting questions to a general practitioner who will triage the question to a specialist is rarely reported in Europe. The already described ask-the-physician services are offered by family physicians or specialists (10,12-14,16-18).

In conclusion, younger age, need for privacy protection, and avoidance of embarrassment at the physician’s office while asking questions related to pregnancy, were all factors independently associated with the utilization of only internet-based medical consultation services without a subsequent visit to a physician. This raises questions about the internet as a supplement in health promotion to young adults.

The results of our study could be used by the medical portals service providers as the basis for possible improvements as they detect the areas of special interest. Future studies should benefit from using a sample representative of the whole population in order to compare characteristics, habits, motives, and other possible factors that distinguish internet users and non-users. Prospective comparative studies could be performed to investigate possible influence of interactive usage of internet for medical consultations on different treatment outcomes. We also suggest exploring the physicians’ experiences and attitudes toward providing health care to patients on the internet as well as their willingness to coach and guide patients toward reliable medical sources and to give their patients a possibility to consult them via e-mail. Such studies should also include the cost-effectiveness analyses.

## 

**Table 5.** Respondent satisfaction with the internet-based medical consultation service ‘Your Questions’ and the obtained answers. Group I (n = 214) – respondents who consulted only internet physician; Group II (n = 822) – respondents who sought internet advice in addition to visiting a physician

**Table:** 

	No. (%) of respondents in			P*
Characteristic of the service	Group I	Group II	overall	
Obtained a specific answer:				
no	17 (7.9)	104 (12.7)	121 (11.7)	0.056
yes	197 (92.1)	718 (87.3)	915 (88.3)	
Was the answer precise?				
no	35 (16.4)	207 (25.2)	242 (23.4)	<0.001
partially	109 (50.9)	286 (34.8)	395 (38.1)	
yes	70 (32.7)	329 (40.0)	399 (38.5)	
Waited too long for an answer:				
no	176 (82.2)	628 (76.4)	804 (77.6)	0.068
yes	38 (17.8)	194 (23.6)	232 (22.4)	
General satisfaction with the answer:				
no	50 (23.4)	327 (39.8)	377 (36.4)	<0.001
yes	164 (76.6)	495 (60.2)	659 (63.6)	

*χ^2^ test.
