# Supplementary material for: Identification of general characteristics, motivation, and satisfaction of internet-based medical consultation service users in Croatia
Source: Croat Med J. 2011 Aug;52(4):557–65. doi: 10.3325/cmj.2011.52.557 (PMC3160707; doi:10.3325/cmj.2011.52.557)
Supplement: Supplementary Table 1 [file CroatMedJ_52_s002.pdf]

Web extra material
Questions in the web survey

1. How many questions have you posted in the section ‘Your questions’ on the internet portal PLIVAzdravlje so far? (one answer only)

2. When posting question(s) in the section ‘Your questions’ on the internet portal PLIVAzdravlje you usually were... (one answer only)
at home
at work
at friends' or relatives' places
at a library, at school or at some other public institution

3. Who did the question you posted concern? (multiple answers possible)
Myself
My partner or spouse
My child
My parent or other relative
Someone else____________

4. Have you or that other person visited the physician before posting the question? (one answer only)
Yes
No

5. Did you try to look for the answer in the articles published on PLIVAzdravlje portal before posting your question? (one answer only)
Yes, I read a couple of articles
Yes, but I did not find anything useful
No, I did not look for any articles at all

6. Did you try to look for the answer in the Q&A repository published on PLIVAzdravlje portal before posting your question? (one answer only)
Yes
No

7. What was your general reason for posting a question in the section ‘Your questions’ on the internet portal PLIVAzdravlje? (multiple answers possible)
I like the opportunity to be able to post a question at any time
I like the privacy I have when posting a question
Physicians are too busy and do not have time to answer to our questions
There are long lines at the physician’s office
I am embarrassed to ask a physician this question
I can’t find time to visit my physician
Other (enter text in text box)

8. What health issue motivated you to post a question in the section ‘Your questions’ on the internet portal PLIVAzdravlje? (multiple answers possible)
I had certain symptoms and wanted to find out what they could be connected to
I needed more information about a certain medical condition.

I wanted a second opinion
I needed more information about a certain drug
I needed more information about a certain diagnostic procedure
I needed more information about a certain medical treatment.
Other (enter text in text box)

9. Did you receive an answer to the question you posted in the section 'Your questions' on the internet portal PLIVAzdravlje? (one answer only)
Yes
No

10. Was the answer you received precise enough? (one answer only)
Yes, absolutely
Partly
No, not exactly
No, not at all

11. Did you have to wait too long to receive the answer? (one answer only)

12. Are you generally satisfied with the answer? (one answer only)
Yes
No, it was too short
No, it had too many complicated words
No, it was too simple compared to the posted question
No - other reason (enter text in text box)

13. After receiving the reply, will you keep searching for more answers about the same issue (one answer only)
Yes, at my physician's office
No, the reply provided me with enough information
Yes I will post a question on another internet page

14. Do you have any suggestions for future enhancements of the interactive service 'Your questions' of the internet portal PLIVAzdravlje? (one answer only)
No
Yes (enter text in text box)

15. What health topics would you like PLIVAzdravlje to address in the future? (free text)

16. Tick your gender
Female
Male

17. What is the level of your education?
Elementary education
Secondary education
Post-secondary education (2 or 3 years)
Post-secondary education (4+ years)

18. Your age? (box for entering a number)

19. What country do you come from? (one answer only)

20. What city do you come from? (one answer only)
